# Molecular diagnostics for the monitoring of the cardiac allograft

**DOI:** 10.1016/j.jhlto.2025.100354

**Published:** 2025-07-25

**Authors:** Lauren K. Truby, Jeffrey Teuteberg

**Affiliations:** a Assistant Professor of Medicine, Associate Medical Director, Adult Heart Transplant Program, Advanced Heart Failure and Transplantation, Department of Medicine, Division of Cardiology, UT Southwestern Medical Center, 5959 Harry Hines Boulevard, Dallas, TX 75390; b Associate Chief of Cardiology, Section Chief of Heart Failure, Cardiac Transplant and Mechanical Circulatory Support, Professor of Medicine, Division of Cardiology, CV 285, Falk Building, 300 Pasteur Drive, Stanford, CA 94305

**Keywords:** Heart transplantation, Transplant rejection, Molecular testing, Biomarkers

## Abstract

Heart transplantation (HT) remains the optimal long-term therapy to improve survival in eligible patients living with end-stage heart disease. Monitoring the health of the allograft over its lifespan is critical to ensure the rapid diagnosis of immunologic, vascular, and myocardial events to enable the timely deployment of appropriate medical therapies. Advances in next-generation sequencing are being applied to screening for allograft rejection to the early detection of malignancy and have already been integrated into the routine care of HT recipients. Further personalization of care by integrating omics and imaging technologies, combined with novel data analysis methods, is at hand.

## Graphical abstract

**Figure:**
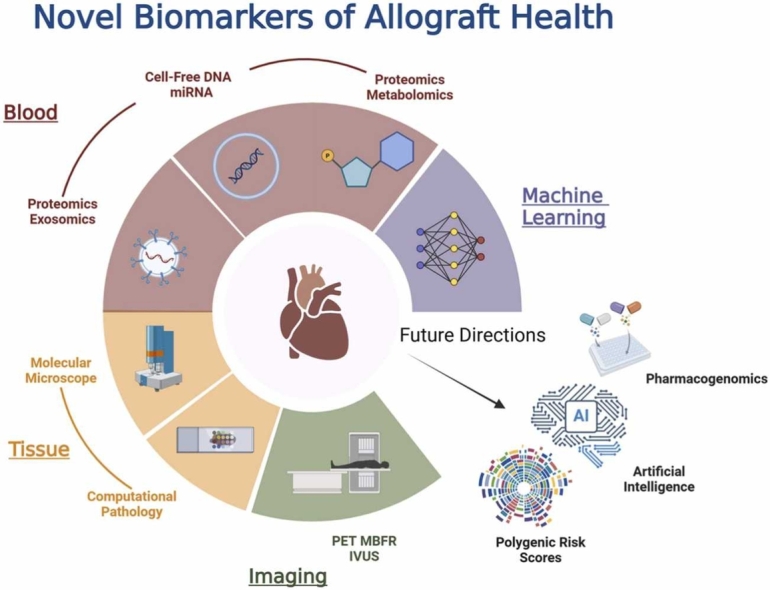

## Introduction

Heart transplantation (HT) remains the optimal long-term therapy to improve survival in eligible patients living with end-stage heart disease. Monitoring the health of the allograft over its lifespan is critical to ensure the rapid diagnosis of immunologic, vascular, and myocardial events to enable the timely deployment of appropriate medical therapies. Advances in next-generation sequencing currently utilized in the screening for allograft rejection are being applied to the early detection of malignancy and have already been integrated into the routine care of HT recipients. Further personalization of care by integrating omics and imaging technologies, combined with novel data analysis methods, is at hand. This review will highlight key opportunities and challenges in utilizing biomarkers to monitor patient and allograft health after HT, including acute rejection, early post-operative events, and to mitigate long-term complications, including cardiac allograft vasculopathy (CAV) and allograft dysfunction. We will highlight current consensus statements on integrating biomarker-based care and review future directions and opportunities to expand the footprint of molecular diagnostics further to improve post-transplant survivorship.

## Molecular diagnostics for the diagnosis of cardiac allograft rejection

### Gene-expression profiling

Extensively reviewed elsewhere, gene-expression profiling (GEP) was the first widely adopted non-invasive molecular diagnostic for acute cellular rejection in HT. In a large, randomized trial compared to endomyocardial biopsy (EMB) in patients predominantly over one year post-transplant, GEP was found to be non-inferior to EMB.^1^ A smaller single-institutional study similarly found GEP was non-inferior to EMB in patients starting two months post-transplant.^2^ Subsequent analyses of a large registry of HT patients managed with GEP found no difference in overall GEP score by race but did demonstrate differential gene expression by race. Despite this, the overall GEP score is utilized clinically and was the first molecular non-invasive rejection surveillance incorporated into management guidelines.^3^, ^4^, ^5^

### Donor-derived cell-free DNA

Extracellular or cell-free DNA (cfDNA) is a highly fragmented, double-stranded DNA molecule of about 150 base pairs thought to be released from nucleosomes during apoptosis. It has a short half-life in the circulation of 30 min to 2 h. However, donor-derived cfDNA (dd-cfDNA) in HT has only recently been widely adopted as a sensitive and specific non-invasive marker of allograft injury and rejection.^6^

The initial experience with dd-cfDNA in HT was in a prospective cohort of 565 samples from 65 HT recipients. Pre-transplant donor and recipient SNP-genotyping was used to distinguish dd-cfDNA and quantify its percentage relative to total circulating cfDNA.^7^ Early elevations of dd-cfDNA returned to background levels one week after HT. Moreover, dd-cfDNA was found to have a sensitivity of 0.58 and specificity of 0.93 for diagnosing acute cellular (ACR) antibody-mediated rejection (AMR) with an AUC of 0.83. The first multicenter study of dd-cfDNA as a molecular diagnostic tool for allograft rejection was d-OAR, an observational, prospective registry with 26 sites collecting 2447 dd-cfDNA samples from 740 HT recipients, 81% of which were during the first post-transplant year.^8^ Median dd-cfDNA levels were significantly higher (0.17% v. 0.07%, p=0.005) with AMR, and dd-cfDNA demonstrated a 97% negative predictive value for AMR at a threshold of 0.2%. The GRAfT (Genome Research Alliance for Transplantation) consortium found dd-cfDNA had a 99% negative predictive for AR value at a threshold of 0.25% with an AUC of 0.92. Moreover, elevations in dd-cfDNA appeared to precede the development of EMB-proven AR and with distinct characteristics when comparing ACR and AMR.

The performance assessments of dd-cfDNA have been mainly based upon correlation with histopathological evidence of rejection. However, GRafT demonstrated a concordance rate of only 77% for ACR and 63% for AMR among pathologists.^9^ Importantly, of the 46 patients with elevated dd-cfDNA without histopathologic evidence of rejection, 59% experienced rejection, allograft dysfunction, or mortality within 1 year of follow-up. In contrast, of those patients with AR on endomyocardial biopsy but without elevations in dd-cfDNA, this composite outcome occurred in only 20%.

### Dual molecular testing

Combining dd-cfDNA with GEP may improve the identification of ACR.^10^ The Surveillance HeartCare Outcomes Registry (SHORE) included 2077 patients with simultaneous GEP and dd-cfDNA testing who were categorized as having dual negative, GEP positive/dd-cfDNA negative, GEP negative/dd-cfDNA positive, or dual positive testing. The incidence of biopsy-proven ACR increased across categories (1.5%, 1.9%, 4.3%, and 9.2%, respectively) with likelihood ratios of ACR 1.37, 2.91, and 3.90 across groups with positive testing, suggesting the two tests yielded complementary data beyond either test alone. When implemented in clinical practice, a single-center study of dual non-invasive molecular testing found that 95% of routine surveillance visits did not result in an EMB. When compared a GEP-alones strategy, which resulted in a higher median number of biopsies(4 (IQR3–5) v. 9 (IQR 8–10), p<0.01) there was no differences in survival free from cellular rejection or graft function at one year.^11^ Notably, 21% of HT recipients in the SHORE registry were African American (AA), a population in which GEP scores differed significantly from those of the non-AA cohort during post-transplant months 2–5, with AA patients having higher scores in the early post-transplant period. There was no difference in median values of dd-cfDNA between AA and non-AA patients, suggesting it is not influenced by race.^12^

Early abnormal dual molecular testing may also presage future adverse events, even in the setting of negative EMB. In SHORE, those with an abnormal GEP and dd-cfDNA and no EMB evidence of rejection had worse freedom from subsequent death or graft dysfunction between 6 months and 2 years as compared to those with either dual negative testing, an abnormal GEP alone or dd-cfDNA alone.^13^ Interestingly, for those who had an episode of biopsy-proven ACR in the first six months post-transplant, as compared to those without rejection, there were no differences in the 2-year freedom from graft dysfunction or death. These data suggest that molecular testing, representing injury across the entire cardiac allograft, may be a better marker of graft health and prognosis than the EMB, which only samples a very small area of the myocardium.

### Mitochondrial dd-cfDNA

Mitochondrial dd-cfDNA is a subset of overall dd-cfDNA that is recognized by the innate immune system as a damage-associated molecular pattern and may act as both a marker and a mediator of tissue injury via its activation of toll-like receptor on antigen-presenting cells.^14^ Dd-cf mitochondrial DNA is higher in Black than White HT recipients starting at 3 months after HT, and this difference persists until at least 1.5 years after the initial surgery. Additional studies are needed to determine if mitochondrial cfDNA may be implicated in racial disparities after HT.^15^

### Circulating micro-RNAs

Micro-RNAs (miRNAs) are small, non-coding RNA molecules that function in RNA silencing and post-transcriptional regulation which have been studied as non-invasive biomarkers of AR with conflicting results.^16^ Early studies in HT found an association between ACR and various miRNAs ^17^ (20). A biosignature for both AMR and ACR comprised of 15 and 11 miRNAs, respectively, provided excellent diagnostic accuracy (AUC 0.92 for ACR and 0.82 for AMR), with external validation of the ACR signature displaying an AUC of 0.91. Unfortunately, a prospective, multi-institutional study including 461 HT recipients with 831 endomyocardial biopsies and 79 discrete rejection episodes could not validate these findings.^18^

### Proteomics, metabolomics, transcriptomics, and exosomics

Other omics technologies have also been investigated as potential novel biomarkers of AR. Non-targeted proteomic profiling was performed using mass spectrometry in 218 consecutive plasma samples from adult HT recipients undergoing routine surveillance EMB.^19^ CD5L, an apoptosis inhibitor that is a key regulator of macrophage activity and inflammation, demonstrated the highest sensitivity and specificity for AR (AUC 0.85) with significant differences across the severity of rejection and a negative predictive value of 94% for ACR ≥ 2R.

Exosomes are membrane-bound vesicles that play critical roles in cell-cell communication by shuttling proteins and genetic material, affecting cellular signaling and regulation.^20^ Kennel et al. identified 15 proteins that distinguished non-AR from ACR and AMR and included proteins reporting on complement activation, immunoglobulin activity, and coagulation.

The IFNy-ELISPOT assay is used to assess the activity of cytokine-producing T cells and IgG-producing B-cells, which may be heightened during periods of allograft rejection.^21^ Clinical Trials of Organ Transplant (CTOT) 5 assessed the association between pre- and post-transplant T cell reactivity and the incidence of ACR defined as ISHLT ≥ 2R. In a cohort of 130 HT recipients, there was not an association between the frequency of alloreactive IFNy-producing peripheral blood mononuclear cells, either as a continuous or dichotomous variable based upon previously published thresholds for AR.

### Tissue-based biomarkers of acute allograft rejection

Much of the last two decades have seen increasing interest in identifying diagnostic biomarkers that could supplant invasive EMB for the surveillance of rejection. However, microarray technology with deep learning analytical methods has been studied as a method to not only improve the diagnostic accuracy of EMB, but to clarify underlying molecular processes in cases of histopathologic ambiguity.^22^ Experience with the Molecular Microscope (Thermofisher, Waltham, MA, USA) has been evaluated in a single-center study of 418 consecutive for-cause endomyocardial biopsies from 237 unique patients.^23^ In cases of microarray-diagnosed AR, dd-cfDNA was more likely to be elevated, and the additional tissue-based archetyping meaningfully changed clinical management in 74% of cases with negative histology. Single-center experiences have also highlighted potential discordance between dd-cfDNA and microarray archetyping, further complicating overall assessments when multiple testing modalities are utilized.^24^

In addition to tissue-based microarray analysis, there is increasing interest in machine learning to provide a computational approach to histologic analysis and improve the accuracy of pathology-based assessments. The Computer-Assisted Cardiac Histologic Evaluation (CACHE) – Grader pipeline^25^ was non-inferior to pathologists and demonstrated nearly identical performance in training and testing sets. Importantly, this digital pathology approach demonstrated excellent sensitivity for high-grade ACR. Another study utilized digital pathology software to annotate 4212 features (1053 normal, 1053 AMR, 1053 healing injury, and 1053 ACR) on 300H&E stained slides.^26^ Within each of the four categories, features were split into testing and training sets, and a deep learning image-based classification method was applied, which was found to have 98% accuracy in the validation set.

In-situ immune profiling using quantitative multiplex immunofluorescence has also been studied to add diagnostic accuracy to assessing the EMB. A single-center study demonstrated that biopsies with high-grade ACR had significantly higher proportions of CD3+, CD8+, and CD68+ cells.^27^ Importantly, FoxP3 and PD-L1 staining differed by rejection grade and clinical severity, suggesting that additional markers beyond immune cell infiltrate may help categorize histopathologic samples with increased diagnostic accuracy.

## Early post-operative allograft monitoring with biomarkers

Beyond non-invasive rejection surveillance, dd-cfDNA has also been used to assess for ischemia-reperfusion injury of the allograft. In one single-center study, early (<6 weeks) dd-cfDNA levels were compared between hearts recovered using temperature-controlled and traditional cold storage methods.^28^ Among 65 donor hearts, 30 were recovered using temperature-controlled storage, which experienced a longer ischemic time (171 v. 212 min, p=0.002) and were from older donors (34 v. 40 years, p=0.04). Temperature-controlled procurement was associated with higher early dd-cfDNA levels, even after adjustment for donor age and prolonged ischemic time. Still, there were no differences in long-term outcomes between recovery platforms.

Metabolomic profiling has also been used to assess allograft health during recovery with *ex-situ* normothermic perfusion devices.^29^ Long-chain acylcarnitines, markers of dysregulated fatty acid oxidation, increased during normothermic perfusion and were associated with elevated lactate and cardiac troponin I levels (36). There were notable differences in the substrate utilization between hearts recovered following brain death and circulatory death, suggesting that differential mechanisms of ischemic injury directly affect cardiac metabolism. Finally, changes in branched-chain amino acids leucine/isoleucine and arginine have been associated with moderate to severe primary graft dysfunction (PGD).

Biomarkers have also been investigated to predict PGD. In pre-transplant proteomic profiling ^30^ CLEC4C, a surface marker of plasmacytoid dendritic cells, was found to be strongly associated with PGD (OR: 3.04, p=2.8×10^−4^) and added incremental predictive capability to a clinical model that included recipient age, creatinine, pre-transplant mechanical circulatory support, donor age, and ischemic time. The authors hypothesize that the activation of PDCs by donor-derived mitochondrial cfDNA, resulting in a surge of pro-inflammatory cytokines, may be a plausible mechanistic link between CLEC4C and PGD.

In addition to innate immunity and inflammation, the complement cascade has also been investigated for potential biomarkers to predict PGD. Proteomic profiling of microvesicles from 88 pre-transplant serum samples was performed using TMT mass spectroscopy.^31^ In this study, lower pre-transplant levels of plasma kallikrein, a critical regulator of the fibrinolytic and kinin systems, were associated with a higher risk of PGD. KLKB1, which converts kininogen into bradykinin, stimulates the release of nitric oxide, increasing vasodilation and vascular permeability. Lower levels may result from kallikrein consumption, which has previously been implicated in other pro-inflammatory states.

Vascular endothelial growth factor (VEGF)-A has also been identified as a novel biomarker of donor PGD risk. A recent study demonstrated that higher levels of donor VEGF-A were associated with more profound myocardial injury as measured by cardiac troponin T and I.^32^ Moreover, donors with high levels of VEGF-A had higher inotrope requirements after transplant. Finally, HT recipients of donors with high VEGF-A levels had a higher incidence of PGD, resulting in more extended length of stay, both overall and in the ICU, as well as an increased risk of renal replacement therapy. Notably, VEGF-A levels increased with donor age, suggesting that it may be a biomarker of overall donor quality and helpful in risk-stratifying extended criteria donors.

## Novel biomarkers of cardiac allograft vasculopathy

The progression of cardiac allograft vasculopathy (CAV) ischemia may result in cellular injury and, thus, elevations of dd-cfDNA. A study of 101 dd-cfDNA samples in 69 patients found the median dd-cfDNA level was not significantly different between patients with and without CAV or across all grades of CAV.^33^ Another study of patients undergoing coronary angiography after the first post-transplant year and a median of 10.9 years post-transplant found no differences in dd-cfDNA between those with and without CAV or those with stable versus progressive CAV.^34^

Given the key role of endothelial dysfunction in the progression of CAV, endothelium-enriched miRNAs were correlated with prevalent CAV in 52 HT recipients undergoing coronary angiography between 5 and 15 years after HT.^35^ Median plasma levels of miR-92a-3p were 1.87-fold higher (p<0.05), and miR-126–3p was 1.94-fold higher (p=0.074) in patients with CAV. When these two miRNAs were combined with the recipient's age and creatinine levels in a multivariable model, the model provided reasonable discrimination between patients with and without CAV (c-statistic 0.8).

The urinary proteome has also been investigated as a potential source of non-invasive biomarkers for CAV.^36^ In a study of capillary electrophoresis coupled with mass spectrometry on urine samples and utilizing machine learning approaches, a proteomic signature for CAV was constructed consisting of 27 proteins clustered into pathways of platelet aggregation, coagulation, cell adhesion, and motility, and demonstrating an AUC of 0.83 and 0.71 in the derivation and validation sets, respectively. The proteomic signature positively correlated with markers of myocardial injury, including high sensitivity troponin T, and inversely correlated with eGFR, even after adjustment for recipient age and sex. A SOMA scan non-targeted proteomic profiling measuring over 1000 proteins was used to quantify their association with CAV.^37^ These proteins cluster into pathways involving inflammation (CST3, NPPB) and platelet activation (F3), which may help to inform research around CAV pathogenesis and identify therapeutic targets for further investigation.

## Novel biomarkers of allograft dysfunction

Clonal hematopoiesis of indeterminant potential (CHIP) refers to the age-related expansion of somatic mutations in hematopoietic stem cells without a hematologic malignancy.^38^ An increasing burden of CHIP has been associated with several cardiovascular diseases, including atherosclerotic coronary artery disease, arrhythmias, heart failure, and stroke.^39^ Studies of CHIP in HT recipients have produced mixed results. In a single-center study, CHIP was not associated with a composite of CAV, graft failure, malignancy, transplantation, and death at a 2% or 5% variant allele frequency after adjustment for age, time from HT to enrollment, hyperlipidemia, and immunosuppression. Another multicenter study found no association between CHIP and CAV, mortality or both either within or across centers.^40^ Lastly, in another study, patients with CHIP had a higher observed mortality rate (aHR: 2.9, CI 1.4 – 6.3, p=0.003) and a higher frequency of CAV grade 2 or 3 (0% v. 18%, p<0.001).^41^ As CHIP is thought, in part, to exert its effects through increased inflammation, some have hypothesized that immunosuppression post-transplant may attenuate some of the cardiovascular risk previously associated with CHIP burden.

Sustained allograft dysfunction after immune-mediate injury is often due to changes in stromal processes in the extracellular matrix that occur as a response to inflammatory insults. This remodeling process is driven by chronic or prior immune-mediated damage and is thought to contribute to the restrictive physiology in those with CAV and prior AR. Using a pathology system that identifies pathologic remodeling in endomyocardial biopsy slides to quantify the degree of injury after HT serially,^42^ 210 stromal features from each biopsy sample were found to identify those that who did and did not experience allograft loss. Fiber density, area ratio of myocytes to the interstitial stroma, fiber solidity, fiber thickness, and fiber orientation was important in distinguishing allograft trajectories and reporting on prior inflammation and immune events.

## Future directions and conclusions

With the rapid evolution of next-generation sequencing technologies and the increasing availability of genomic data, the next decade will likely see a surge in biomarker discovery to fuel precision medicine approaches to donor-recipient matching, personalization of immunosuppressive therapies, and noninvasive diagnostic modalities. With an amassing of genetic data in HT recipients, we will likely see the identification of pharmacogenomic variants to guide treatment strategies and predict adverse events and polygenic risk scores for post-transplant outcomes.^43^, ^44^ Concomitantly, the applications of machine learning approaches are likely to increase, including leveraging large datasets from electronic health records to predict post-transplant outcomes and facilitate early therapeutic interventions. Machine learning algorithms also have the potential to improve donor-recipient matching, increase the accuracy of survival predictions, and provide opportunities to optimize post-transplant pharmacotherapies.^45^ Integrating biomarker-guided and personalized approaches to care, validated in randomized controlled trials, represents the next frontier in heart transplantation.

## Declaration of competing interest

The authors declare the following financial interests/personal relationships which may be considered as potential competing interests: Jeffrey Teuteberg reports a relationship with Abbott that includes: consulting or advisory. Jeffrey Teuteberg reports a relationship with AbioMed Inc that includes: consulting or advisory. Jeffrey Teuteberg reports a relationship with Broadview Ventures Inc that includes: consulting or advisory. Jeffrey Teuteberg reports a relationship with CareDx Inc that includes: consulting or advisory and speaking and lecture fees. Jeffrey Teuteberg reports a relationship with CVRx Inc that includes: consulting or advisory. Jeffrey Teuteberg reports a relationship with Medtronic Inc that includes: consulting or advisory. Jeffrey Teuteberg reports a relationship with Paragonix Technologies, Inc that includes: speaking and lecture fees. Jeffrey Teuteberg reports a relationship with Takeda Pharmaceuticals USA Inc that includes: consulting or advisory. AHA 23CDA1050881, LKT NIH K23HL171828, LKT If there are other authors, they declare that they have no known competing financial interests or personal relationships that could have appeared to influence the work reported in this paper.
